# Drug resistance and genotyping studies of *Salmonella* Enteritidis isolated from broiler chickens in Iran

**DOI:** 10.3389/fvets.2025.1542313

**Published:** 2025-02-18

**Authors:** Mohammad Reza Piryaei, Seyed Mostafa Peighambari, Jamshid Razmyar

**Affiliations:** Department of Avian Diseases, Faculty of Veterinary Medicine, University of Tehran, Tehran, Iran

**Keywords:** antimicrobial resistance, broiler chicken, poultry, *Salmonella* Enteritidis, virulence factors

## Abstract

**Introduction:**

Poultry products are considered an important source of *Salmonella* infections. Transmission of non-typhoidal *Salmonella enterica* serovars to humans has been a great concern worldwide. Occurrence of multi-drug resistance, adding to the presence of various virulence factors, which facilitate the pathogenesis of *Salmonella*, would cause tremendous risk for both human and animals’ health.

**Methods and results:**

During 2023, out of a total number of 1,274 samples from broilers in Iran, 114 isolates of *Salmonella* spp. (8.94%) were detected from which 97 isolates were confirmed as *Salmonella* Enteritidis (SE). Eight virulence genes including *inv*A, *sef*A, *sop*E, *spv*C, *hil*A, *agf*A, *siv*H and *lpf*A, were detected among SE isolates and it was found that all isolates harbored these genes at the rate of 100% except for *spv*C, which was present in 96.90% of the SE isolates. In phenotypic evaluation of resistance against 16 antimicrobial agents, high resistance rates were observed against nalidixic acid, ampicillin, amoxicillin–clavulanate and ciprofloxacin. While resistance to tetracycline, streptomycin and chloramphenicol was found to be moderate, it was very low to azithromycin, sulfamethoxazole-trimethoprim, amikacin, gentamicin, ceftriaxone and cefotaxime. However, all isolates were sensitive to meropenem, ceftazidime and aztreonam. The mean of MAR index values was 0.26 and 72.15% of the isolates were found to be highly resistant. In detection of 14 resistance genes among SE isolates, five genes including *bla*TEM, *tet*A, *tet*B, *sul*1 and *str*A/B were found with prevalence rates of 63.92, 36.08, 61.85, 10.30 and 14.43%, respectively.

**Discussion:**

The high prevalence rates of MDR in SE, along with the overwhelming presence of major virulence factors raise public health concerns. These data highlight the great potential risks of the presence and transmission of highly pathogenic MDR *Salmonella* to humans from chicken meat sources, as well as the need for more effective surveillance for antimicrobial use in the poultry industry. Reducing/optimizing the use of antimicrobials, improving poultry management procedures, using probiotics and biosecurity or vaccines are essential to deal with this issue.

## 1 Introduction

*Salmonella*, a Gram negative, motile bacterium from the *Enterobacteriaceae* family, is a primary pathogen which can infect a wide range of animals. As a zoonotic disease, salmonellosis has been one of the most common causes of gastroenteritis and food poisoning in humans in recent years affecting most parts of the world including developed countries (1). Salmonellosis in humans has been reported from almost all countries and the rates of occurrence vary, but usually it has not been decreased significantly in the last few years, even in well-developed countries (2). For example, in 2010, in the United States the incidence of salmonellosis was higher than any other food-borne pathogens (17.6 infections /100,000 population) (3). In that year, the World Health Organization (WHO) reported 153 million cases of *Salmonella* gastroenteric infections worldwide. Additionally, in 2019, WHO estimated 26 million cases of *Salmonella* gastroenteritis and 118,000 deaths globally (4).

Poultry products are a major reservoir of *Salmonella*, posing risks to human health, poultry production and food products (5, 6). *Salmonella* spreads vertically through eggs and horizontally via direct or indirect contact, persisting on farms for extended periods (7). Poultry salmonellosis causes high mortality, reduced flock performance, and increased susceptibility to other diseases leading to economic losses (8). Surveillance programs worldwide aim to control *Salmonella* and reduce its entry into the food chain (9). In the U.S., foodborne salmonellosis costs an estimated $4–11 billion annually in medical care, lost production, and premature deaths (10).

In poultry production, using antimicrobial agents is very common for different purposes such as growth promotion, prophylactic and control of infections (11). However, antimicrobial usage contributes to development of drug resistance; and posing risks to public health, the poultry industry, and the environment (12). It was estimated that in 2019, around five million people around the world died because of antimicrobial resistance (13). In addition to the pathogenic potential of *Salmonella*, these bacteria can develop resistance to several antimicrobials, which may make medical treatment of the infections even more challenging (12, 14). Antimicrobial resistant bacteria can transmit resistance either vertically to their progeny or horizontally to other bacterial populations through mobile genetic elements; thereby, facilitating the dissemination of resistance (13–15).

As a primary pathogen, *Salmonella* is equipped with many virulence properties. Every virulence property may play a distinct role in the complex pathogenicity, the ability to survive, and/or transmission of the bacteria. The genes which encode these virulence properties are integrated into the plasmid or chromosomal genome and their expressions and interactions are yet to be well-understood (16, 17). Chromosomal virulence-associated genes helping *Salmonella* with its attack and invasion capabilities include *inv*A, *hil*A and *siv*H which are essential for the intrusion of epithelial cells (18). *Salmonella* effector protein attached by *sop*E gene help *Salmonella* in the disorganizing host cell membrane (19). The aggregative fimbria, agf operon, takes part in an essential interaction of *Salmonella* with the digestive tract cells of the host and facilitates microbial self-aggregation for higher rates of survival (20). The *Salmonella*-encoded fimbria (sef operon), encodes the major subunit of the fimbrial protein SEF14 which supports interaction between the organisms and the macrophages of the host (21). The plasmid-mediated *spv*C gene counts liable for vertical transmission of *Salmonella* through eggs (22). Long polar fimbria (*lpf* operon), is a plasmid-mediated virulence factor which encodes an important part of a fimbria and is associated with the fascination of the organism for Peyer’s patches and its attachment to intestinal M cells (20).

Among more than 2,500 recognized *Salmonella* serovars, about 10% are found in poultry, with *Salmonella* Enteritidis (SE) and *S.* Typhimurium (ST) being the most prevalent worldwide (23–25). *Salmonella* Enteritidis infections in humans are often linked to the consumption of contaminated poultry products; especially eggs, while *S.* Typhimurium infections are mostly associated with the consumption of pork, poultry, beef and even seafood (26–28). Given the public health and economic impact of salmonellosis, along with its complex pathogenesis, studying virulence genes and antimicrobial resistance in poultry-origin *Salmonella* is very critical. This study was conducted to provide data and updates on poultry-origin *Salmonella* from Iran, in order to achieve a better understanding for control and treatment of salmonellosis.

## 2 Materials and methods

### 2.1 Sample collection and bacteriological procedures

**Figure 1:**
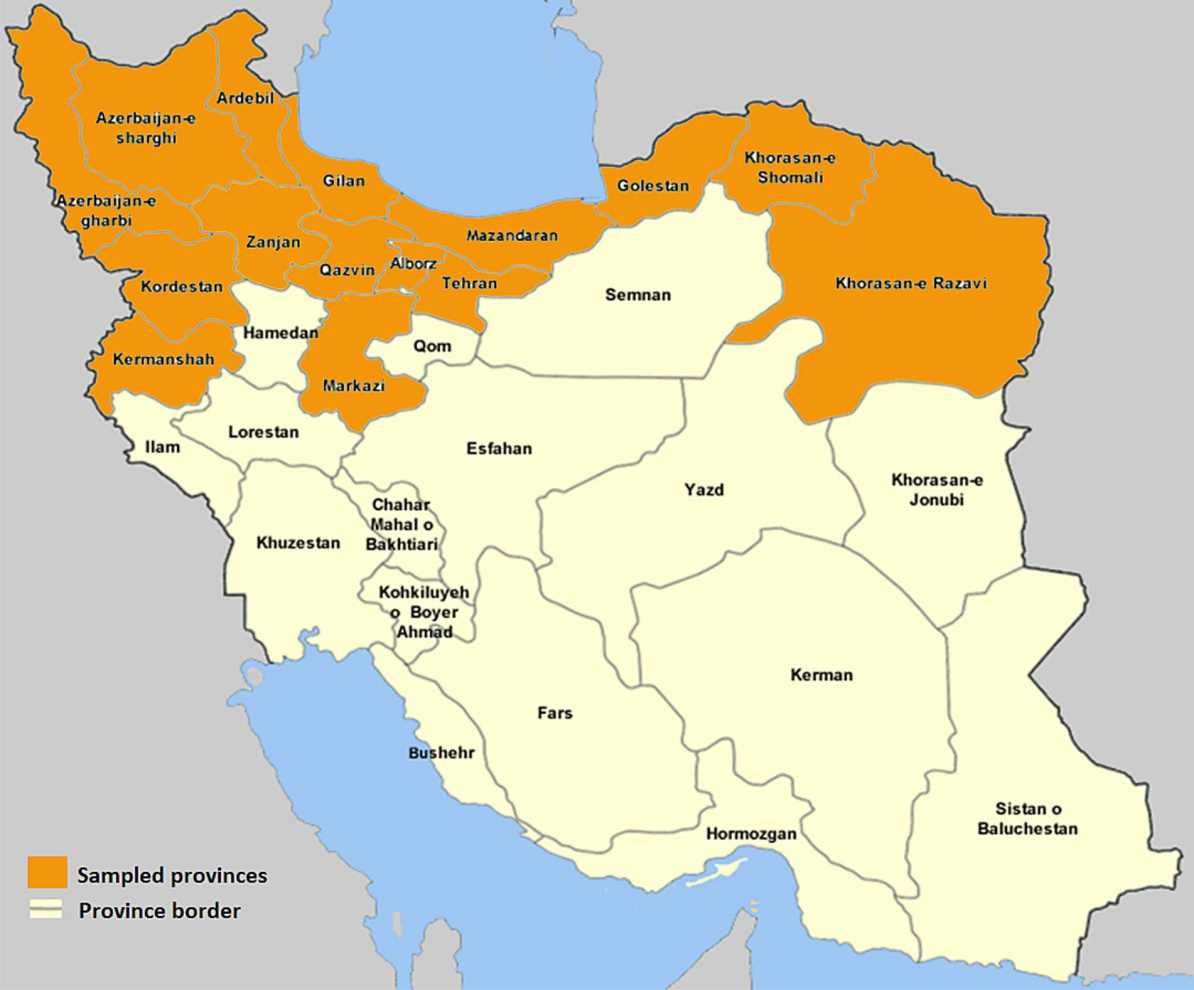
Map of Iran, the sampled areas are marked in orange.

This cross-sectional study was completed in 2023. The geographical regions from which the samples were received are shown in Figure 1. The sampled provinces have a high density of poultry farms. Our laboratory and our collaborative laboratories in Tehran city often receives samples from those flocks for *Salmonella* isolation. The population of broiler chickens in the sampling areas was estimated to be more than 1,000,000,000 birds in 2023. Sterile cotton swabs contained in 10 mL of Selenite F as enrichment medium were used for swabbing from every submitted sample from the broiler chicken flocks. Samples were collected aseptically and brought to the microbiology laboratory in an insulated icebox. *Salmonella* isolation and identification were carried out according to standard procedures previously described (29). Briefly, samples were inoculated onto selenite F enrichment broth at 41°C for 24 h, followed by sub-cultivation on *Salmonella*-*Shigella* (SS) and MacConkey agar at 37°C for 24 h. Typical black-centered colonies on SS and colorless colonies on MacConkey plates were picked and subsequently cultured onto nutrient agar plate (NA; Oxoid, UK). The biochemical confirmation was done by using triple sugar iron (TSI), motility indole urea (MIU), catalase and oxidase tests.

### 2.2 Antimicrobial susceptibility test

The susceptibility of the SE isolates to a panel of 16 antimicrobial agents was determined by the agar disk diffusion method and the interpretation of results was carried out according to the National Committee for Clinical Laboratory Standards guidelines (30). The antimicrobial agents were selected because of their importance in human and veterinary medicine. The evaluated antimicrobials and their concentrations were: amikacin, AN (30 μg), gentamicin, GM (10 μg), streptomycin, S (10 μg), meropenem, MEN (10 μg), ceftriaxone, CRO (30 μg), cefotaxime, CTX (10 μg), ceftazidime, CAZ (30 μg), aztreonam, AZT (30 μg), amoxicillin–clavulanate, AMC (30 μg), ampicillin, AM (10 μg), azithromycin, AZM (15 μg), ciprofloxacin, CP (5 μg), nalidixic acid, NA (30 μg), sulfamethoxazole-trimethoprim, SXT (1.25–23.75 μg), tetracycline, TE (10 μg), chloramphenicol, C (30 μg). All antibacterial disks were provided from Padtan Teb Co (Tehran, Iran). The ATCC reference strains *Escherichia coli* ATCC 25922, *Pseudomonas aeruginosa*, ATCC 27853, and *E. coli* ATCC 35218 were used for quality control purposes. In this study, the SE isolates with intermediate susceptibility classification were considered to be resistant to that drug and multi-resistance was defined as resistance to 3 or more classes of antibacterials.

### 2.3 Multiple antibiotic resistance indexing

Multiple antibiotic resistance (MAR) indexing has been considered a suitable and valid method for tracking the source of bacteria. To calculate the MAR index, the number of resistant antibiotics for an organism would be divided by the total number of antibiotics to which the organism has been exposed. MAR index values larger than 0.2 indicate high resistance of the organism, where antibiotics are often used. The multiple antibiotic resistance (MAR) index of all isolates was calculated and the results were interpreted using a proven method as described previously (31).

### 2.4 Confirmation of *Salmonella* genus and *Salmonella* Enteritidis by PCR

To extract bacterial DNA, 1 mL pure overnight culture of each SE isolate grown overnight at 37°C for 16 h was transferred to a clean 1.5 mL microtube and centrifuged for 5 min at 10,000×*g*. The supernatants were carefully removed and discarded. The pellet was re-suspended in 300 μL sterile double distilled water by vortexing, incubated for 15 min at 100°C, chilled on ice immediately, and centrifuged again for 5 min at 14,000×*g* at 4°C. The supernatant was removed and used as template DNA. The concentration of DNA was determined by Biophotometer (Eppendorff, Germany) and adjusted to approximately 200 ng for each PCR reaction. The supernatant was stored at −20°C for further use.

In this study, *invA* gene specific primers were used to confirm the *Salmonella* genus (Table 1). Also, in order to identify serovar Enteritidis one pair of specific primers for amplification of *sdf-*ι gene were used (Table 1). All primers were synthesized by Bioneer (South Korea). Amplification reactions for *Salmonella* genus and serovar Enteritidis confirmation were carried out in a 25 μL reaction volume containing 12.5 μL of 2x mastermix (*Taq* 2x Red Master Mix, Ampliqon, Denmark), 0.5 μL each of forward and reverse primers (10 pmol/μL), 2 μL of DNA template, and 9.5 μL nuclease-free water. *Salmonella* Enteritidis PT21 strain (32) and dH2O (instead of template DNA) were used as positive and negative controls, respectively, in all PCR reaction sets. Amplifications were programmed in a thermocycler (SensoQuest, Germany) as described below. For *Salmonella* genus, 95°C for 1 min followed by 38 cycles of 95°C for 30 s, 64°C for 30 s, 72°C for 30 s, and a final extension at 72°C for 4 min was used (33). For serovar Enteritidis, program was as follows: 95°C for 2 min followed by 30 cycles of 95°C for 60 s, 57°C for 60 s, 72°C for 2 min, and a final extension at 72°C for 5 min (34). The amplified products were detected by gel electrophoresis in 1.5% agarose gel containing Safe Stain^®^ (SinaClon) at 100 V for 30 min in 1x TAE buffer and visualized under UV illumination. A 100 bp DNA ladder (Yekta Tajhiz Azma, Iran) was used as a molecular weight marker for the PCR products in gel electrophoresis.

**Table 1:** Targeted genes, primer sequences and expected amplicon sizes for identification of *Salmonella* genus and *Salmonella* Enteritidis.

PCR	Target gene	Primer sequence (5′-3′)	Amplicon size (bp)	References
Detection of *Salmonella* genus	*inv*A	F-GTGAAATTATCGCCACGTTCGGGCAA R-TCATCGCACCGTCAAAGGAACC	284	(33)
Detection of Serovar Enteritidis	*sdf-*ι	F-TGTGTTTTATCTGATGCAAGAGG R-CGTTCTTCTGGTACTTACGATGAC	293	(34)

### 2.5 Detection of virulence genes

All isolates were examined for the presence of seven important virulence genes namely: *hil*A, *agf*A, *lpf*A, *siv*H, *sef*A, *sop*E and *spv*C. Each of seven virulence genes was amplified by using specific primer pairs and according to the PCR protocols described in Table 2. The preparation of reaction mixtures and gel electrophoresis were done as described above. The positive control (SE PT21 strain) and negative control (dH2O instead of template DNA) were used in all PCR reaction sets for validation. All primers of virulence genes were synthetized by SinaClon (Tehran, Iran).

**Table 2:** Targeted genes, primer sequences and amplicon sizes for detection of virulence factors.

Target gene	Primer sequence (5′-3′)	Amplicon size (bp)	References
*agf*A	F-TCCACAATGGGGCGGCGGCG R-CCTGACGCACCATTACGCTG	350	(98)
*lpf*A	F-CTTTCGCTGCTGAATCTGGT R-CAGTGTTAACAGAAACCAGT	250	(99)
*hil*A	F-CTGCCGCAGTGTTAAGGATA R-CTGTCGCCTTAATCGCATGT	497	(100)
*siv*H	F-GTATGCGAACAAGCGTAACAC R-CAGAATGCGAATCCTTCGCAC	763	(101)
*sef*A	F-GATACTGCTGAACGTAGAAGG R-GCGTAAATCAGCATCTGCAGTAGC	488	(102)
*sop*E	F-GGATGCCTTCTGATGTTGACTGG R-ACACACTTTCACCGAGGAAGCG	398	(103)
*spv*C	F-CCCAAACCCATACTTACTCTG R-CGGAAATACCATCTACAAATA	669	(104)

### 2.6 Detection of antimicrobial resistance genes

All isolates were screened by using PCR to investigate the presence of 14 antimicrobial resistance genes including seven *β*-lactamase genes (*bla*TEM, *bla*SHV, *bla*OXA, *bla*CTX-M-1, *bla*CTX-M-2, *bla*CTX-M-9 and *bla*CTX-Mg8/25), three tetracycline resistant genes (*tet*A, *tet*B and *tet*C), three sulfonamide resistant genes (*sul*1, *sul*2 and *sul*3) and one streptomycin resistant gene (*str*A/B). For the detection of β-lactam genes, two cycles of multiplex PCR were carried out as described previously (35). Multiplex PCR were also performed to detect the resistance genes for sulfonamides (*sul*1, *sul*2 and *sul*3), tetracycline (*tet*A, *tet*B and *tet*C), and a single PCR for streptomycin (*str*A/B) according to the methods described previously (36). The PCR reaction mixture preparations and gel electrophoresis were done as described above. The specific primers used to detect antimicrobial resistance genes were synthetized by SinaClon (Table 3). More details about the PCR procedures are provided in Supplementary Table S1.

**Table 3:** Targeted genes, primer sequences and expected amplicon sizes for detection of antimicrobial resistance genes.

PCR	Target gene	Primer sequence (5′-3′)	Amplicon size (bp)	References
Multiplex PCR-1	*bla*TEM	F-CCGTTCATCCATAGTTGCCTGAC R-TTTCCGTGTCGCCCTTATTC	800	(35)
	*bla*SHV	F-AGCCGCTTGAGCAAATTAAAC R-ATCCCGCAGATAAATCACCAC	713	
	*bla*OXA	F-GGCACCAGATTCAACTTTCAAG R-GACCCCAAGTTTCCTGTAAGTG	564	
Multiplex PCR-2	*bla*CTX-M-1	F-TTAGGAARTGTGCCGCTGYA R-CGATATCGTTGGTGGTRCCAT	688	
	*bla*CTX-M-2	F-CGTTAACGGCACGATGAC R-CGATATCGTTGGTGGTRCCAT	404	
	*bla*CTX-M-9	F-TCAAGCCTGCCGATCTGGT R-TGATTCTCGCCGCTGAAG	561	
	*bla*CTX-Mg8/25	F-AACRCRCAGACGCTCTAC R-TCGAGCCGGAASGTGTYAT	326	
Multiplex PCR-3	*sul*1	F-CGGCGTGGGCTACCTGAACG R-GCCGATCGCGTGAAGTTCCG	433	(36)
	*sul*2	F-CGGCATCGTCAACATAACCT R-TGTGCGGATGAAGTCAGCTC	721	
	*sul*3	F-CAACGGAAGTGGGCGTTGTGGA R-GCTGCACCAATTCGCTGAACG	244	
Multiplex PCR-4	*tet*A	F-GGCGGTCTTCTTCATCATGC R-CGGCAGGCAGAGCAAGTAGA	502	(36)
	*tet*B	F-CGCCCAGTGCTGTTGTTGTC R-CGCGTTGAGAAGCTGAGGTG	173	
	*tet*C	F-GCTGTAGGCATAGGCTTGGT R-GCCGGAAGCGAGAAGAATCA	888	
Uniplex PCR	*str*A/B	F-ATGGTGGACCCTAAAACTCT R-CGTCTAGGATCGAGACAAAG	893	(36)

### 2.7 Statistical analysis

The attained data on antimicrobial susceptibility was presented in Excel worksheets (MS-2017). The prevalence was calculated using descriptive analysis.

## 3 Results

### 3.1 Bacteriology

Out of 1,274 samples that were cultured, 114 (8.94%) *Salmonella* isolates were recovered and from which 97 (85.09%) isolates were identified to be *Salmonella* Enteritidis. The remaining 17 (14.91%) *Salmonella* isolates were excluded from this study.

### 3.2 Virulence factors

**Figure 2:**
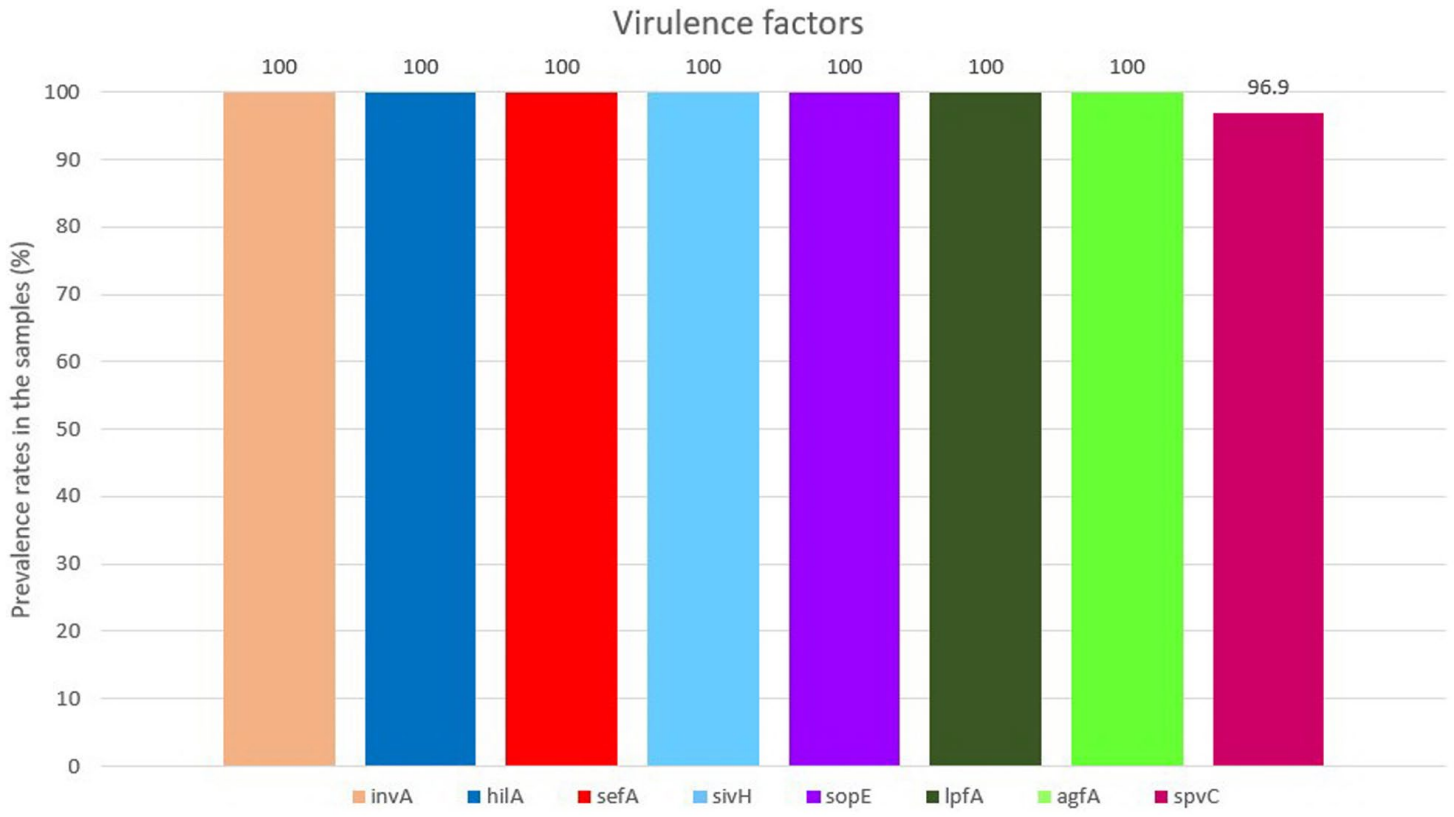
Diagram of virulence factor patterns among the isolates.

All isolates were positive for the presence of all eight virulence genes, except for *spv*C gene which was found in 94 (96.90%) out of 97 isolates (Figure 2). For detailed information, refer to Supplementary Table S2.

### 3.3 MAR index and phenotypic resistance profiles

The antimicrobial susceptibility evaluation of 16 antimicrobial agents belonging to 10 different classes of antimicrobials revealed that 100% of isolates showed resistance to nalidixic acid, followed by 83.5 and 80.41% resistance observed to ampicillin and amoxicillin–clavulanate, respectively. In addition, 79.38% of isolates showed resistance to ciprofloxacin. Moderate to low resistance rates were found against tetracycline (38.14%), streptomycin (12.37%) and chloramphenicol (11.3%). All isolates were 100% sensitive to meropenem, ceftazidime and aztreonam and a high sensitivity was observed to azithromycin (95.88%), sulfamethoxazole-trimethoprim (96.91%), amikacin (97.94%), gentamicin (97.94%), ceftriaxone (97.94%) and cefotaxime (98.97%). Details are given in Supplementary Table S3.

The calculated mean of MAR index for all isolates was 0.26, the lowest MAR index was 0.06 and the highest MAR index was 0.5. Considering MAR indexes above 0.2 are highly resistant, the prevalence of these isolates was 72.15%. The most prevalent antimicrobial resistance pattern (24.74%) was AMC, AM, CP, NA (MAR index = 0.25). Resistance patterns and MAR indexes are shown in Table 4.

**Table 4:** Multiple antibiotic resistance (MAR) index and resistance patterns.

Resistance patterns	MAR index	Patterns prevalence (%)	MAR index prevalence (%)
NA	0.06	4.12	4.12
CP, NA	0.12	3.1	12.37
AM, NA		4.12	
AMC, NA		5.15	
S, CP, NA	0.18	1.03	11.34
AMC, AM, NA		6.18	
AM, CP, NA		3.1	
AMC, CP, NA		1.03	
AMC, AM, CP, NA	0.25	24.74	28.86
AMC, CP, NA, TE		1.03	
AM, CP, NA, TE		2.06	
CRO, AMC, CP, NA		1.03	
AMC, AM, CP, NA, TE	0.31	20.62	28.86
AM, CP, NA, TE, C		2.06	
S, AMC, AM, CP, NA		3.1	
AMC, AM, NA, TE, C		1.03	
AMC, AM, AZM, CP, NA		1.03	
AMC, AM, CP, NA, C		1.03	
S, AMC, AM, CP, NA, TE	0.37	3.1	7.20
AMC, AM, CP, NA, TE, C		2.06	
AMC, AM, AZM, CP, NA, C		1.03	
CRO, CTX, AMC, AM, CP, NA		1.03	
S, AMC, AM, CP, NA, SXT, TE	0.43	1.03	4.10
AN, AMC, AM, AZM, CP, NA, TE		1.03	
GM, S, AMC, AM, CP, NA, C		1.03	
AN, GM, AMC, AM, CP, NA, TE		1.03	
S, AMC, AM, CP, NA, SXT, TE, C	0.5	2.06	3.10
S, AMC, AM, AZM, CP, NA, TE, C		1.03	

Amikacin (AN), Amoxicillin–clavulanate (AMC), Ampicillin (AM), Azithromycin (AZM), Cefotaxime (CTX), Ceftriaxone (CRO), Chloramphenicol (C), Ciprofloxacin (CP), Gentamicin (GM), Nalidixic acid (NA), Streptomycin (S), Sulfamethoxazole-trimethoprim (SXT), Tetracycline (TE).

### 3.4 Genotypic resistance patterns

Among 14 investigated resistance genes, only five genes including *bla*TEM, *tet*A, *tet*B, *sul*1 and *str*A/B were variably detected among isolates. No resistance genes were found in 11 isolates, 10 of which had a MAR index below 0.2. In four isolates which had MAR indexes above 0.4, all five genes of *bla*TEM, *tet*A, *tet*B, *sul*1 and *str*A/B were simultaneously present. The most frequent detected resistance gene was *bla*TEM (63.92%), followed by *tet*B (61.85%), *tet*A (36.08%), *str*A/B (14.43%) and *sul*1 (10.30%). The related details are given in Supplementary Table S3.

## 4 Discussion

Different serovars of *Salmonella* have been detected among various poultry sources with a large variation in prevalence. For instance, only in poultry egg samples, studies around the world have reported *Salmonella* prevalence of 0% in Egypt (37), 0.3% in Bangladesh (38), 2.9% in Eastern Ethiopia (39), 3% in Belgium (40), 3.3% in North India (41), 5.4% in China (42), 7.7% in South India (43), 24.17% in Nigeria (44) and 34% in Spain (45). In Iran, a *Salmonella* contamination rate of 3.8% (46) and 6.3% (47) in poultry eggs have been indicated. Additionally, Bahramianfard and co-workers (47) found a SE prevalence of 1.3 and 2.3% in egg and poultry samples, respectively. These variations in *Salmonella* prevalence could be related to differences in geographical regions, periods of sampling, hygienic measures and management programs.

The importance of *Salmonella* Enteritidis among non-typhoid serovars is undoubtable and unquestionable. In 2005, Velge et al. ranked *Salmonella* Enteritidis as the most prevalent serovar in poultry and since then, it has been confirmed by many studies performed in different regions of the world (48). Results from 37 countries revealed prevalence rates from 19.2% (in Cameroon) to 49% (in Tunisia) in Africa, and 5 to 93.7% in Europe and Asia (3, 47, 49).

Many researchers in Iran have conducted studies on *Salmonella* prevalence in Iranian poultry flocks. Zahraei Salehi et al. found 30 (15.6%) *Salmonella* isolates in 192 samples taken from liver and intestine of broiler chickens (50). The prevalence of *Salmonella* in broiler chicken carcasses in abattoir was reported to be 8.3% (51). In 2011–2012, 25.8% of the fecal samples originated from poultry slaughterhouses were positive for *Salmonella*, from which 40.4% of the isolates were identified as SE (52). In another study, Afshari et al. found 14 (14%) *Salmonella* isolates among 100 samples taken from poultry, in which six (43%) isolates were confirmed as serovar Enteritidis (53). Recent studies on *Salmonella* contamination of poultry meat samples provided from retail stores in Iran have reported the prevalence of *Salmonella* to be 8.75% in 80 samples (54) or 2.7% in 150 samples (55). Even though the geographical regions of the latter studies were not included in our study, our results are in harmony with these findings. This high prevalence is correlated with the complexity of the transmission of *Salmonella*. This complexity could be explained by the ability to survive in different environments, the vast spectrum of hosts and carriers, being equipped with different virulence factors and resistance against antimicrobial agents (56–60). These results confirm the point that new hygienic measures and protocols should be implemented to control this zoonosis pathogen.

We found all eight detected virulence genes at very high rates in SE isolates including genes of *Salmonella* pathogenicity islands 1 and 2 (SPI-1 and SPI-2), genes related to cell adhesion and genes which play important roles in pathologic mechanisms. It could be interpreted from previous studies that among non-typhoid serovars, the presence of investigated virulence factors in SE was higher compared to other common serovars such as ST. For instance, in another study with the same framework on poultry-origin *Salmonella* from wet markets in Bangladesh, Siddiky et al. detected the presence of eight virulence genes including *inv*A, *agf*A, *lpf*A, *hil*A, *siv*H, *sef*A, *sop*E and *spv*C with the rate of 100% presence in SE isolates (20). Our results are compatible with the results of Siddiky et al. except for *spv*C, a plasmid-mediated gene. We found *spvC* in 96.90% of SE isolates compared with that of in 100% (20, 61), 92% (62) and 88.6% (63) of SE isolates in previous studies (20, 61). These variations in the results could be related to the geographical distribution of the strains and also to the developments and optimizations made over the years in molecular methods and materials.

The *sef*A gene is associated with the production of an SE fimbrial protein with a molecular mass of 17 kDa (SEF 17), which inhibits the binding of the extracellular matrix protein named fibronectin to SE (64). The 100% prevalence of *sef*A gene among SE isolates of this study confirmed the previous findings and as some researchers have suggested, the *sef*A gene can be considered a proper candidate for identification of serovar Enteritidis (20, 61–63). Aside from the *inv*A gene role in cell invasion, the *sef*A gene plays an important role in diagnosis of *Salmonella* species (65, 66). Additionally, for invasion into the host’s cells, expression of the *hil*A gene also increases the virulence of *Salmonella*, and this gene is usually 100% present in SE isolates (67, 68). Both *inv*A and *hil*A genes could be considered as symbols of the invasive nature of *Salmonella* (20, 61, 62). The 100% presence of *lpf*A, *agf*A, and *sop*E genes among SE isolates of the present study is consistent with the findings of previous investigations (20, 64, 69). The *agf*A gene takes part in the development of biofilm, which is crucial for the survival of the organism and, due to its relation to coding a fimbrial protein, it is liable for cell adhesion (70). Previous findings also confirm our data (20, 61, 70), except the results of Borges and co-workers in Brazil, which found *agf*A gene only in 96% of SE isolates from chickens (62). In addition, the 100% prevalence of *sop*E gene –encoded in SPI-1- is accordant to previous studies (20, 62, 71, 72). The high rate of virulence genes detected in this study, reinforces the necessity of practical hygienic measures to control and reduce *Salmonella* infections, considering the vigorous invasive nature of this pathogen. The synergism between virulence genes and antimicrobial resistance could escalate the risk of infection and its consequences, and facilitate the spread of resistant pathogenic *Salmonella* in human and animals (71, 73, 74).

In this study, we evaluated the recent situation of antimicrobial resistance among *Salmonella* Enteritidis isolated from broiler chickens from both phenotypic and genetic aspects. Phenotypically, SE isolates were found to have high resistance rates against nalidixic acid (100%), ampicillin (83.5%), amoxicillin–clavulanate (80.41%), and ciprofloxacin (79.38%); moderate resistance to tetracycline (38.14%), and low resistance to streptomycin (12.37%), and chloramphenicol (11.34%). In comparing these results to other studies in Iran, Bahramianfard et al. (47) observed resistance to nalidixic acid (87.3%), kanamycin (25.4%), colistin sulphate (23.8%) and trimethoprim-sulfamethoxazole (20.6%) among SE isolates from poultry meat and egg samples. In another study, Khademi et al. also reported high rates of antimicrobial resistance to *Salmonella* serovars recovered from clinical samples in Iran (1983–2019) including resistance to tetracycline (54.3%), ceftizoxime (50.6%), streptomycin (50.2%), and nalidixic acid (48.1%) (75). In a study by Vaez et al., *Salmonella* isolates from animals were mostly resistant to nalidixic acid (67%), tetracycline (66.9%), streptomycin (49.6%), and trimethoprim-sulfamethoxazole (41.6%) (76). Moreover, Besharati et al. showed higher resistance rates against antimicrobial agents among *Salmonella* serovars originated from poultry processed meat compared to those of obtained from human stool samples (77). The maximum resistance rates among *Salmonella* isolates from poultry processed meat were as follows tetracycline (59%), trimethoprim-sulfamethoxazole (43%), azithromycin (42%), chloramphenicol (27%); while the resistance rates were significantly lower in human stool samples indicating tetracycline (13.6%), trimethoprim-sulfamethoxazole (9.1%), azithromycin (9.1%), and chloramphenicol (0%) (77). Nemati and Ahmadi reported the antimicrobial resistance rates among *Salmonella* isolates from western regions of Iran as such ampicillin (100%), nalidixic acid (73.13%), trimethoprim-sulfamethoxazole (58.20%), streptomycin (47.76%), and tetracycline (43.28%) (78).

In other countries, in China, *Salmonella* isolates recovered from abattoirs was shown to be resistant mostly against nalidixic acid (99.5%), ampicillin (87.8%), tetracycline (51.9%), ciprofloxacin (48.7%), and trimethoprim-sulfamethoxazole (48.1%) (79). In Bangladesh, Parvin et al. found a high resistance among *Salmonella* isolates originated from frozen chicken meat samples against oxytetracycline (100%), trimethoprim-sulfamethoxazole (89.2%), tetracycline (86.5%), nalidixic acid (83.8%), amoxicillin (74.3%), and pefloxacin (74.3%) (80). In a Chinese study, it was demonstrated that *Salmonella* isolates recovered from hatcheries were highly resistant to ciprofloxacin (77%), sulfisoxazole (73%), and ampicillin (55.6%) (81). Furthermore, in another study from Bangladesh, Siddiky et al. detected high resistance levels against streptomycin (100%), ciprofloxacin, tetracycline and gentamicin (80%); moderate resistance to amikacin, amoxicillin–clavulanate, azithromycin and sulphamethoxazole-trimethoprim (20%) in SE isolated from broilers (20). In comparison, Siddiky and coworkers detected 100% resistance against ciprofloxacin and streptomycin, 86.66% resistance to tetracycline, nalidixic acid and gentamicin, 66.66% resistance to ampicillin and 40% resistance against amoxicillin–clavulanate in ST isolates from broiler chickens (20). Our other findings from antimicrobial susceptibility test results indicated low resistance levels against azithromycin (4.12%), sulfamethoxazole-trimethoprim (3.09%), ceftriaxone, gentamicin and amikacin (2.06%) and cefotaxime (1.03%) and full sensitivity against meropenem, ceftazidime and aztreonam that are comparable with those of from other studies (20).

A high MAR index (> 0.2) indicates a frequent use of antibiotics suggesting poultry products as a high-risk source for multi-drug resistant (MDR) *Salmonella* strains. In addition, Mishra et al. indicated that MAR index values >0.2 were associated with a high-risk source of contamination and MAR > 0.4 indicated a fecal source of contamination (82). In this study, isolates had a mean MAR index of 0.259 with the highest MAR of 0.5. Seven out of 97 (7.21%) isolates had MAR index values of >0.4 and 70 (72.16%) isolates had a MAR index value of >0.2. Moreover, we detected 28 different resistance patterns and the most prevalent pattern (20.62%) was resistance to amoxicillin–clavulanate, ampicillin, ciprofloxacin, nalidixic acid, and tetracycline. Moreover, one finding that drew our attention was that resistance to sulfamethoxazole-trimethoprim that was observed only in isolates showing MAR index values of >0.43. Strict surveillances should be applied in the regions where isolates with high MAR index values were detected.

We investigated the presence of seven ESBL genes among our isolates. Only one, the *bla*TEM gene, was present in 63.92% of isolates, and it was the most prevalent gene. Other *β*-lactamase resistance genes were not detected in any of isolates from this study. In this regard, Sales et al. detected *bla*TEM gene in 34.61%, and *bla*SHV in 11.53% of ST isolates from children with diarrhea, and a total rate of 57.69% of their isolates were positive for ESBL (83). According to Lai et al., 89.9% of 129 *Salmonella* isolated from fecal samples of pigs, goats, cattle, rabbits, chickens and ducks between September 2016 and May 2019 in China possessed *β*-lactamase resistance genes and the *bla*TEM gene was detected in 82.9% of those isolates. Other β-lactamase genes including *bla*OXA (20.2%), *bla*CTX-M (6.2%), and *bla*CMY (2.3%) were also detected (84). Furthermore, Das et al. found a high prevalence of *bla*TEM (95.4%) in *Salmonella* isolated from broiler flocks of Bangladesh (85). In Bangladesh, Siddiky et al. also detected *bla*TEM gene in 73.3% of *Salmonella* isolated from broiler chickens (86) and 62.06 to 69.62% of *Salmonella* isolated from different parts of wet markets (20). In Iraq, Hassan et al. detected *bla*TEM in 52.6% of *Salmonella enterica* isolates from 28 broiler chicken farms, with no trace of *bla*SHV, *bla*CTX-M, and *bla*OXA (87). In contrast, Ramatla et al. detected *bla*TEM in 7% of ST and 28% of SE isolates from chickens and rats in layer farms of South Africa. Among SE isolates, they also detected other ESBL genes including *bla*CTX-M (39%), *bla*CTX-M1 (44%), and *bla*CTX-M9 (33%). The total number of ESBL encoding genes was higher in ST compared to SE isolates (88). Moreover, Hardiati et al. identified a 100% presence of *bla*TEM in *Salmonella* isolates from chicken farms of Java, Indonesia (89).

In evaluating the prevalence of antimicrobial resistance genes related to tetracyclines, we detected *tet*A gene in 36.08% and *tet*B in 61.85% of the isolates. No *tet*C positive isolate was detected in any isolates. Moreover, the *str*A/B gene, which is related to resistance against streptomycin was identified in 14.43% of SE isolates. Similarly, a lower prevalence of 10.30% for sulphonamide-related resistance gene *sul*1 was found in SE isolates. No isolates harbored *sul*2 and *sul*3 resistance genes. Four (4.12%) isolates possessed all of these five detected genes with the genotypic pattern of “*bla*TEM, *tet*A, *tet*B, *sul*1, *str*A/B,” while in 11 (11.34%) isolates no resistance gene was detected at all. Based on our findings, it was interesting that the mean of MAR index values for those five isolates with the mentioned genotypic resistance pattern was 0.465, while the mean of MAR index values for the 11 isolates with no detected resistance gene was 0.126. This significant difference in the latter results can confirm the correlation and harmony between the phenotypic and genotypic resistance results. Keeping in mind that modulation of gene expression could depend on several different factors, it is a nonnegligible point, and it can justify the higher prevalence of some resistance genes, compared to phenotypic resistance rates against related classes of antimicrobial drugs (90).

The given data in the previous paragraph are comparable to those of Siddiky et al. study (86). They found prevalence values of 100% for *tet*A and 20% for *sul*1 and *str*A/B in *Salmonella* isolates from chicken samples provided from wet markets in Bangladesh (86). Moreover, Das et al. detected the *tet*A, *tet*B, and *tet*C genes in 81.4, 19.8, and 10.47% of *Salmonella* isolates in commercial broiler farms of Bangladesh, respectively. In addition, 37.2% of their isolates harbored the *sul*1 gene (85). Consequently, Hardiati et al. identified *tet*A in 33.3% of the *Salmonella* isolates from chicken farms in Indonesia (89). Wang et al. detected *tet*A, *bla*TEM, *sul*1, and *sul*2 genes with prevalence rates of 81.3, 62.5, 25, and 100%, respectively, among *Salmonella* isolated from retail meat samples in China (91). Recently, Nazari Moghadam et al. reported the presence of tetracycline-resistant (*tet*A, *tet*B, *tet*C, *tet*G) and sulphonamide-resistant (*sul*1, *sul*2, and *sul*3) genes in 100, 23, 27, 39% for *tet*A-*tet*G and 84, 50, and 17% for *sul*1-*sul*3, respectively, among *Salmonella* isolated from poultry meat (54). Our genotypic resistance patterns were also consistent with earlier findings in Iran and other countries.

It is important to note the fact that this study performed on samples received from only 15 out of 31 Iranian provinces and; moreover, due to different regional antimicrobial use practices, the interpretation and generalizability of our findings may have some limitations. Working on additional samples from other provinces may help for a better understanding of SE distribution among broiler chicken population in Iran and of the relevant genetic data. The variability in regional antimicrobial use practices, including varying levels of antibiotic stewardship, accessibility, and usage policies, creates discrepancies in the selection pressure exerted on bacterial populations (92). These variations can result in significant regional differences in MAR index values, making it challenging to extrapolate our findings to broader populations or global settings. To address these challenges, we emphasize the importance of adopting standardized sampling protocols and incorporating regional antibiotic usage data to provide a more comprehensive understanding of AMR trends. Despite these limitations, our study contributes valuable insights into the resistance patterns of *Salmonella* and underscores the need for harmonized methodologies to enable better cross-regional comparisons and the development of targeted mitigation strategies.

The observed resistance patterns in *Salmonella* isolates may result from factors beyond direct antibiotic use. Horizontal gene transfer in shared environments, such as farms or processing facilities, could facilitate the spread of resistance genes among bacterial populations. Environmental contamination with antibiotics or residues may create sub-lethal selection pressures, favoring resistant strains (93). Additionally, historical antibiotic use and regional differences in host immune responses or ecological factors may contribute to resistance dynamics (94). Methodological variations, including sampling protocols and resistance classification criteria, could also influence the results. To better understand these patterns, future studies should integrate environmental, genetic, and methodological data alongside comprehensive antimicrobial use surveillance, enabling more targeted and effective mitigation strategies.

To control AMR in *Salmonella*, vaccination and stricter biosecurity measures are essential. Vaccination reduces *Salmonella* prevalence and minimizes the need for antibiotics by enhancing immunity. Multivalent and live-attenuated vaccines should be integrated into routine poultry health programs, with regular monitoring to assess their effectiveness. Also, There have been several trials to develop a vaccine against SE (95). Stricter biosecurity measures, including enhanced hygiene, restricted farm access, pest control, and clean feed and water management, prevent *Salmonella* introduction and spread. Implementing all-in/all-out flock systems further reduces cross-contamination (96). Combining these strategies with robust antimicrobial stewardship and surveillance can effectively reduce *Salmonella* infections and mitigate the spread of AMR in poultry systems.

To effectively mitigate AMR, we propose implementing several targeted policies. First, non-therapeutic antibiotic use should be banned, with therapeutic applications allowed only under strict veterinary supervision. Vaccination programs targeting key *Salmonella* serovars should be mandated to lower infection rates and reduce reliance on antibiotics, supported by financial incentives to encourage adoption. Enhanced biosecurity measures, including improved hygiene, pest control, and controlled access to farms, should be enforced through regular inspections. Additionally, a centralized surveillance system to monitor antibiotic usage and resistance trends in poultry farming is crucial for guiding evidence-based interventions. Training programs for farmers and veterinarians on AMR, sustainable practices, and biosecurity protocols should be prioritized to build compliance and awareness. Finally, farms that adopt sustainable, antibiotic-free practices should receive financial incentives, while penalties for non-compliance will ensure accountability. These combined measures can significantly reduce *Salmonella*-related AMR risks and promote sustainable poultry farming practices.

Whole genome sequencing (WGS) surpasses classical PCR in detecting virulence and antimicrobial resistance (AMR) genes by providing comprehensive genomic analysis. Unlike PCR, which targets specific genes, WGS identifies both known and novel genes, their genetic context (e.g., plasmids or mobile elements), and mutations linked to resistance. It enables full characterization of gene clusters and regulatory elements, offering deeper insights into pathogen biology and gene transfer mechanisms. Additionally, WGS is scalable for epidemiological studies and outbreak tracking, making it a more powerful and versatile tool than PCR for studying virulence and AMR (97).

## 5 Conclusion

Data obtained from this study highlights the great potential risks of the presence and transmission of highly pathogenic MDR *Salmonella* to humans from chicken meat sources as well as the need for more effective surveillance program for antibiotic use in the poultry industry. Strict hygiene and sanitation standards in poultry product chains should be reprogrammed to reduce transmission. High associations among the virulence genes, phenotypic resistance and genotypic resistance were evident in the results. The high prevalence rates of MDR in *Salmonella* Enteritidis isolates, along with the overwhelming presence of major virulence factors raise public health concerns. Therapeutic, preventive and imprudent uses of antimicrobial agents have resulted in the exposure of pathogens to these drugs and increasing the risk of developing resistance. Reducing/optimizing the use of antimicrobials, improving poultry management procedures, strict clean and disinfection measures, proper use of probiotics and biosecurity—especially considering the possibility of using vaccines—are essential to deal with this issue.

To address antimicrobial resistance in *Salmonella*, we recommend studying molecular mechanisms like plasmids and efflux pumps, tracking resistance trends through longitudinal studies, and exploring the role of horizontal gene transfer and environmental reservoirs. Omics-based research can identify resistance determinants and therapeutic targets, while investigations into host-microbiota interactions and non-antibiotic interventions, such as vaccines and probiotics, can support sustainable mitigation strategies. These efforts are essential for effective AMR control.

## Data Availability

The original contributions presented in the study are included in the article/Supplementary material, further inquiries can be directed to the corresponding author.

## Supplementary material

SUPPLEMENTARY TABLE S1 Thermal profiles of the studied genes.

SUPPLEMENTARY TABLE S2 Laboratory codes, isolation dates and details of virulence factors results.

SUPPLEMENTARY TABLE S3 Origin of the isolates, phenotypic results, patterns of drug resistance, MAR index and genotypic results of resistance genes.
